# (5*Z*)-5-(2-Hydroxy­benzyl­idene)-2-thioxo-1,3-thia­zolidin-4-one methanol hemisolvate

**DOI:** 10.1107/S1600536809039555

**Published:** 2009-10-03

**Authors:** Durre Shahwar, M. Nawaz Tahir, Muhammad Asam Raza, Bushra Iqbal, Sana Naz

**Affiliations:** a Department of Chemistry, Government College University, Lahore, Pakistan; b Department of Physics, University of Sargodha, Sargodha, Pakistan

## Abstract

In the title compound, C_10_H_7_NO_2_S_2_·0.5CH_3_OH, the dihedral angle between the aromatic rings is 11.43 (11)° and a short intra­molecular C—H⋯S contact occurs. The methanol solvent mol­ecule is equally disordered over two sets of sites. In the crystal, inversion dimers linked by pairs of N—H⋯O hydrogen bonds occur. The methanol solvent mol­ecule connects the dimers through O—H⋯S and O—H⋯O inter­molecular hydrogen bonds. Further stability is afforded by C—H⋯π and π–π inter­actions [centroid–centroid separation = 3.5948 (13) Å].

## Related literature

For related structures, see: Barreiro *et al.* (2007 ▶); Delgado *et al.* (2006 ▶). For graph-set notation, see: Bernstein *et al.* (1995 ▶).
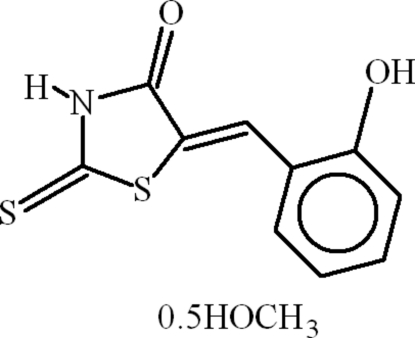


## Experimental

### 

#### Crystal data

C_10_H_7_NO_2_S_2_·0.5CH_4_O
*M* *_r_* = 253.33
Monoclinic, 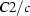

*a* = 20.4859 (16) Å
*b* = 6.4422 (4) Å
*c* = 18.4377 (15) Å
β = 108.724 (4)°
*V* = 2304.5 (3) Å^3^
*Z* = 8
Mo *K*α radiation
μ = 0.45 mm^−1^
*T* = 296 K
0.28 × 0.15 × 0.12 mm

#### Data collection

Bruker Kappa APEXII CCD diffractometer
Absorption correction: multi-scan (*SADABS*; Bruker, 2005 ▶) *T*_min_ = 0.925, *T*_max_ = 0.947
11803 measured reflections
2636 independent reflections
1562 reflections with *I* > 2σ(*I*)
*R*_int_ = 0.040

#### Refinement

*R*[*F* ^2^ > 2σ(*F* ^2^)] = 0.044
*wR*(*F* ^2^) = 0.101
*S* = 1.03
2636 reflections
167 parameters
8 restraints
H atoms treated by a mixture of independent and constrained refinement
Δρ_max_ = 0.30 e Å^−3^
Δρ_min_ = −0.28 e Å^−3^

### 

Data collection: *APEX2* (Bruker, 2007 ▶); cell refinement: *SAINT* (Bruker, 2007 ▶); data reduction: *SAINT*; program(s) used to solve structure: *SHELXS97* (Sheldrick, 2008 ▶); program(s) used to refine structure: *SHELXL97* (Sheldrick, 2008 ▶); molecular graphics: *ORTEP-3* (Farrugia, 1997 ▶) and *PLATON* (Spek, 2009 ▶); software used to prepare material for publication: *WinGX* (Farrugia, 1999 ▶) and *PLATON*.

## Supplementary Material

Crystal structure: contains datablocks global, I. DOI: 10.1107/S1600536809039555/hb5115sup1.cif

Structure factors: contains datablocks I. DOI: 10.1107/S1600536809039555/hb5115Isup2.hkl

Additional supplementary materials:  crystallographic information; 3D view; checkCIF report

## Figures and Tables

**Table 1:** Hydrogen-bond geometry (Å, °)

*D*—H⋯*A*	*D*—H	H⋯*A*	*D*⋯*A*	*D*—H⋯*A*
N1—H1*N*⋯O2^i^	0.86	1.95	2.811 (2)	173
O1—H1*O*⋯O11^ii^	0.82	1.95	2.735 (7)	159
O1—H1*O*⋯O11^iii^	0.82	2.06	2.871 (7)	169
O11—H11⋯S2^i^	0.84 (3)	2.80 (5)	3.317 (8)	122 (4)
C6—H6⋯S1	0.93	2.57	3.264 (3)	132
C4—H4⋯*Cg*2^iv^	0.93	2.81	3.599 (3)	143

Symmetry codes: (i) 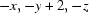; (ii) 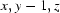; (iii) 
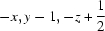
; (iv) 
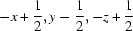
. *Cg*2 is centroid of the C1–C6 benzene ring.

**Fig. 1.**
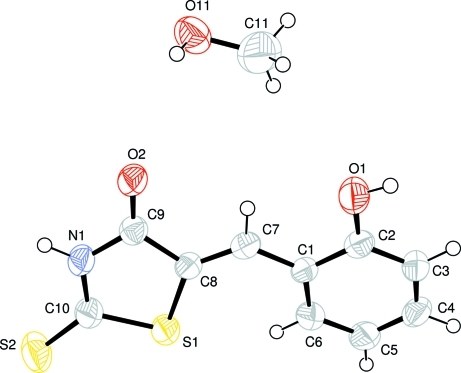
View of (I) with displacement ellipsoids drawn at the 50% probability level. H-atoms are shown by small circles of arbitrary radius.

**Fig. 2.**
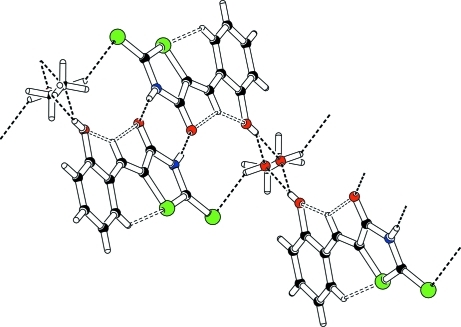
The partial packing of (I), which shows that molecules form dimers and dimers are connected to each other with the help of methanol by intermolecular H-bondings. The double dotted lines represent intramolecular H-bondings.

## supplementary crystallographic information

### Comment

The title compound (I, Fig. 1) has been prepared owing to medicinal properties of rhodanine derivatives.

The crystal structure of (II) (*Z*)-5-(2-Fluorobenzylidene)-2-thioxothiazolidin-4-one (Delgado *et al.*, 2006) and (III) 5-(2-Hydroxybenzylidene)-2-thioxo-1,3-thiazolidin-4-one dimethylsulfoxide solvate (Barreiro, *et al.*, 2007) have been published. The title compound (I) differs from (III) due to solvate i.e methanol instead of dimethylsulfoxide.

The title molecule basically consits of dimers due to intermolecular H-bondings of N—H···O type with *R*_2_^2^(8) ring motifs (Bernstein *et al.*, 1995). There exist a strong interamolecular H-bonding of C—H···S type and two weak intramolecular H-bondings of C–H···O (Table 1, Fig. 2) forming a twisted S(6) and two planar S(5) ring motifs. In (I), the 2-Hydroxybenzylidene moiety A (C1—C7/O1) and the rhodanine moiety B (C8/C9/N1/C10/S1/S2/O2) are planar with maximum r.m.s. deviations of 0.0042 and 0.0044 Å, respectively from their mean square planes. The dihedral angle between A/B is 11.35 (10)°. The methanol solvent of crystallization connects the dimers through O—H···S and O—H···O intermolecular H-bondings and form another ring motif *R*_3_^3^(10) (Fig. 2). The molecules are stabilized in the form of two dimensional polymeric networks due to C—H···π interaction (Table 1) and π–π interactions between the centroids of heterocyclic ring *Cg*1 (C8/C9/N1/C10/S1) and the benzene ring *Cg*2 (C1—C6). The distance between *Cg*1···*Cg*2^i^ [symmetry code i = *x*, 1 + *y*, *z*] and *Cg*2—*Cg*1^ii^ [symmetry code ii = *x*, -1 + *y*, *z*] is 3.5948 (13) Å.

### Experimental

Rhodanine (0.266 g, 0.2 mol), salicylaldehyde (0.244 g, 0.2 mol) and K_2_CO_3_ (0.553 g, 0.4 mol) were dissolved in 10 ml distilled water at room temperature. The stirring was continued for 24 h and reaction was monitored by TLC. The precipitates were formed during neutalization of the reaction mixture with 5% HCl. The precipitates were filtered off and washed with saturated solution of NaCl. The crude material obtained was recrystalized in methanol to affoard light red needles of (I).

### Refinement

The multiplicity factor of C and O atom of methanol was intially refined and later it was fixed to 0.5.

The coordinates of H-atom of hydroxy group were refined. The coordinates of H-atoms of methanol were also refined with constraints. The H-atoms were positioned geometrically with N—H = 0.86, C—H = 0.93 Å for aromatic like H atoms and constrained to ride on their parent atoms, with *U*_iso_(H) = xUeq(C, N, O), where *x* = 1.5 for methyl H-atoms and *x* = 1.2 for all othe H atoms.

### Crystal data

**Table:**

C_10_H_7_NO_2_S_2_·0.5CH_4_O	*F*(000) = 1048
*M**_r_* = 253.33	*D*_x_ = 1.460 Mg m^−^^3^
Monoclinic, *C*2/*c*	Mo *K*α radiation, λ = 0.71073 Å
Hall symbol: -C 2yc	Cell parameters from 2636 reflections
*a* = 20.4859 (16) Å	θ = 2.1–27.5°
*b* = 6.4422 (4) Å	µ = 0.45 mm^−^^1^
*c* = 18.4377 (15) Å	*T* = 296 K
β = 108.724 (4)°	Cut needle, light red
*V* = 2304.5 (3) Å^3^	0.28 × 0.15 × 0.12 mm
*Z* = 8	

### Data collection

**Table:**

Bruker Kappa APEXII CCD diffractometer	2636 independent reflections
Radiation source: fine-focus sealed tube	1562 reflections with *I* > 2σ(*I*)
graphite	*R*_int_ = 0.040
Detector resolution: 7.50 pixels mm^-1^	θ_max_ = 27.5°, θ_min_ = 2.1°
ω scans	*h* = −22→26
Absorption correction: multi-scan (*SADABS*; Bruker, 2005)	*k* = −5→8
*T*_min_ = 0.925, *T*_max_ = 0.947	*l* = −23→22
11803 measured reflections	

### Refinement

**Table:**

Refinement on *F*^2^	Primary atom site location: structure-invariant direct methods
Least-squares matrix: full	Secondary atom site location: difference Fourier map
*R*[*F*^2^ > 2σ(*F*^2^)] = 0.044	Hydrogen site location: inferred from neighbouring sites
*wR*(*F*^2^) = 0.101	H atoms treated by a mixture of independent and constrained refinement
*S* = 1.03	*w* = 1/[σ^2^(*F*_o_^2^) + (0.0357*P*)^2^ + 1.2743*P*] where *P* = (*F*_o_^2^ + 2*F*_c_^2^)/3
2636 reflections	(Δ/σ)_max_ < 0.001
167 parameters	Δρ_max_ = 0.30 e Å^−^^3^
8 restraints	Δρ_min_ = −0.28 e Å^−^^3^

### Special details

**Table:**

Geometry. Bond distances, angles *etc*. have been calculated using the rounded fractional coordinates. All su's are estimated from the variances of the (full) variance-covariance matrix. The cell e.s.d.'s are taken into account in the estimation of distances, angles and torsion angles
Refinement. Refinement of *F*^2^ against ALL reflections. The weighted *R*-factor *wR* and goodness of fit *S* are based on *F*^2^, conventional *R*-factors *R* are based on *F*, with *F* set to zero for negative *F*^2^. The threshold expression of *F*^2^ > σ(*F*^2^) is used only for calculating *R*-factors(gt) *etc*. and is not relevant to the choice of reflections for refinement. *R*-factors based on *F*^2^ are statistically about twice as large as those based on *F*, and *R*- factors based on ALL data will be even larger.

### Fractional atomic coordinates and isotropic or equivalent isotropic displacement parameters (Å^2^)

**Table:**

	*x*	*y*	*z*	*U*_iso_*/*U*_eq_	Occ. (<1)
S1	0.15938 (4)	0.58242 (10)	−0.00444 (4)	0.0600 (2)	
S2	0.14589 (4)	0.94002 (13)	−0.11023 (5)	0.0802 (3)	
O1	0.06021 (9)	0.1385 (3)	0.18770 (11)	0.0619 (7)	
O2	0.01091 (8)	0.7729 (2)	0.06117 (9)	0.0516 (6)	
N1	0.06936 (9)	0.8655 (3)	−0.02055 (10)	0.0467 (7)	
C1	0.14189 (11)	0.2372 (3)	0.12922 (12)	0.0389 (7)	
C2	0.12016 (12)	0.0955 (3)	0.17442 (12)	0.0428 (8)	
C3	0.15870 (12)	−0.0790 (4)	0.20299 (13)	0.0485 (8)	
C4	0.21827 (13)	−0.1171 (4)	0.18715 (13)	0.0531 (9)	
C5	0.24140 (13)	0.0198 (4)	0.14359 (14)	0.0566 (9)	
C6	0.20350 (13)	0.1944 (4)	0.11522 (13)	0.0512 (9)	
C7	0.10043 (11)	0.4192 (3)	0.10120 (12)	0.0422 (8)	
C8	0.10408 (11)	0.5631 (3)	0.05020 (12)	0.0396 (7)	
C9	0.05620 (11)	0.7391 (3)	0.03308 (12)	0.0405 (8)	
C10	0.12115 (12)	0.8120 (4)	−0.04789 (13)	0.0502 (8)	
O11	−0.0100 (5)	0.8280 (6)	0.2318 (3)	0.066 (3)	0.500
C11	0.014 (2)	0.6235 (11)	0.262 (3)	0.112 (14)	0.500
H1N	0.04538	0.97578	−0.03643	0.0561*	
H1O	0.04906	0.03909	0.20888	0.0743*	
H3	0.14403	−0.17151	0.23327	0.0582*	
H4	0.24344	−0.23670	0.20598	0.0637*	
H5	0.28232	−0.00571	0.13348	0.0680*	
H6	0.21932	0.28673	0.08585	0.0614*	
H7	0.06521	0.44009	0.12207	0.0507*	
H11	−0.035 (3)	0.804 (7)	0.1868 (16)	0.0792*	0.500
H11A	−0.012 (4)	0.500 (7)	0.244 (5)	0.1677*	0.500
H11B	0.019 (4)	0.608 (13)	0.316 (4)	0.1677*	0.500
H11C	0.058 (3)	0.572 (11)	0.265 (5)	0.1677*	0.500

### Atomic displacement parameters (Å^2^)

**Table:**

	*U*^11^	*U*^22^	*U*^33^	*U*^12^	*U*^13^	*U*^23^
S1	0.0676 (4)	0.0634 (4)	0.0594 (4)	0.0280 (4)	0.0349 (4)	0.0186 (3)
S2	0.0750 (5)	0.0963 (6)	0.0837 (6)	0.0304 (4)	0.0457 (4)	0.0451 (5)
O1	0.0609 (11)	0.0550 (11)	0.0805 (13)	0.0130 (9)	0.0376 (10)	0.0213 (9)
O2	0.0495 (10)	0.0516 (10)	0.0591 (11)	0.0152 (8)	0.0249 (9)	0.0124 (8)
N1	0.0446 (12)	0.0464 (11)	0.0511 (12)	0.0133 (9)	0.0180 (10)	0.0116 (10)
C1	0.0446 (13)	0.0359 (12)	0.0364 (13)	0.0055 (10)	0.0131 (10)	−0.0019 (10)
C2	0.0448 (13)	0.0401 (13)	0.0433 (13)	0.0031 (11)	0.0137 (11)	−0.0050 (11)
C3	0.0596 (16)	0.0406 (13)	0.0448 (14)	0.0038 (12)	0.0162 (12)	0.0050 (11)
C4	0.0631 (17)	0.0431 (14)	0.0490 (15)	0.0180 (12)	0.0124 (13)	0.0002 (12)
C5	0.0598 (16)	0.0600 (16)	0.0560 (16)	0.0212 (13)	0.0269 (13)	0.0061 (13)
C6	0.0589 (16)	0.0516 (15)	0.0481 (15)	0.0113 (13)	0.0243 (12)	0.0067 (12)
C7	0.0425 (13)	0.0409 (13)	0.0439 (13)	0.0037 (10)	0.0148 (11)	−0.0049 (11)
C8	0.0422 (13)	0.0374 (12)	0.0387 (12)	0.0049 (10)	0.0123 (10)	−0.0002 (11)
C9	0.0385 (13)	0.0410 (13)	0.0394 (13)	0.0029 (11)	0.0087 (11)	−0.0007 (11)
C10	0.0483 (14)	0.0550 (15)	0.0468 (15)	0.0131 (12)	0.0146 (12)	0.0081 (12)
O11	0.082 (7)	0.067 (2)	0.049 (5)	−0.007 (2)	0.022 (5)	−0.0010 (19)
C11	0.16 (3)	0.063 (3)	0.15 (3)	−0.004 (7)	0.10 (2)	0.002 (8)

### Geometric parameters (Å, °)

**Table:**

S1—C8	1.745 (2)	C2—C3	1.378 (3)
S1—C10	1.744 (3)	C3—C4	1.365 (4)
S2—C10	1.622 (3)	C4—C5	1.375 (4)
O1—C2	1.356 (3)	C5—C6	1.372 (4)
O2—C9	1.219 (3)	C7—C8	1.340 (3)
O1—H1O	0.8200	C8—C9	1.466 (3)
O11—C11	1.45 (2)	C3—H3	0.9300
O11—H11	0.84 (3)	C4—H4	0.9300
N1—C9	1.373 (3)	C5—H5	0.9300
N1—C10	1.357 (3)	C6—H6	0.9300
N1—H1N	0.8600	C7—H7	0.9300
C1—C7	1.443 (3)	C11—H11A	0.96 (6)
C1—C2	1.402 (3)	C11—H11B	0.97 (9)
C1—C6	1.395 (4)	C11—H11C	0.95 (8)
			
C8—S1—C10	92.67 (11)	O2—C9—C8	126.50 (19)
C2—O1—H1O	109.00	O2—C9—N1	123.54 (19)
C11—O11—H11	104 (4)	S1—C10—S2	124.08 (16)
C9—N1—C10	118.3 (2)	S2—C10—N1	126.4 (2)
C10—N1—H1N	121.00	S1—C10—N1	109.52 (17)
C9—N1—H1N	121.00	C4—C3—H3	120.00
C2—C1—C7	118.7 (2)	C2—C3—H3	120.00
C2—C1—C6	117.4 (2)	C3—C4—H4	120.00
C6—C1—C7	123.9 (2)	C5—C4—H4	120.00
O1—C2—C3	122.6 (2)	C6—C5—H5	120.00
O1—C2—C1	116.96 (19)	C4—C5—H5	120.00
C1—C2—C3	120.4 (2)	C1—C6—H6	119.00
C2—C3—C4	120.5 (2)	C5—C6—H6	119.00
C3—C4—C5	120.6 (2)	C1—C7—H7	115.00
C4—C5—C6	119.3 (3)	C8—C7—H7	115.00
C1—C6—C5	121.8 (2)	O11—C11—H11A	123 (6)
C1—C7—C8	130.9 (2)	O11—C11—H11B	113 (6)
S1—C8—C9	109.51 (15)	O11—C11—H11C	123 (6)
C7—C8—C9	120.2 (2)	H11A—C11—H11B	98 (8)
S1—C8—C7	130.34 (18)	H11A—C11—H11C	98 (7)
N1—C9—C8	109.96 (19)	H11B—C11—H11C	97 (8)
			
C10—S1—C8—C7	179.8 (2)	C2—C1—C7—C8	−170.5 (2)
C10—S1—C8—C9	0.0 (2)	C6—C1—C7—C8	10.7 (4)
C8—S1—C10—S2	179.12 (17)	O1—C2—C3—C4	179.1 (2)
C8—S1—C10—N1	0.05 (18)	C1—C2—C3—C4	−0.5 (3)
C10—N1—C9—O2	−179.9 (2)	C2—C3—C4—C5	1.2 (4)
C10—N1—C9—C8	0.1 (3)	C3—C4—C5—C6	−0.8 (4)
C9—N1—C10—S1	−0.1 (3)	C4—C5—C6—C1	−0.1 (4)
C9—N1—C10—S2	−179.14 (18)	C1—C7—C8—S1	2.0 (4)
C6—C1—C2—O1	180.0 (2)	C1—C7—C8—C9	−178.2 (2)
C6—C1—C2—C3	−0.4 (3)	S1—C8—C9—O2	179.96 (19)
C7—C1—C2—O1	1.1 (3)	S1—C8—C9—N1	−0.1 (2)
C7—C1—C2—C3	−179.3 (2)	C7—C8—C9—O2	0.1 (3)
C2—C1—C6—C5	0.7 (3)	C7—C8—C9—N1	−179.90 (19)
C7—C1—C6—C5	179.5 (2)		

### Hydrogen-bond geometry (Å, °)

**Table:**

*D*—H···*A*	*D*—H	H···*A*	*D*···*A*	*D*—H···*A*
N1—H1N···O2^i^	0.86	1.95	2.811 (2)	173
O1—H1O···O11^ii^	0.82	1.95	2.735 (7)	159
O1—H1O···O11^iii^	0.82	2.06	2.871 (7)	169
O11—H11···S2^i^	0.84 (3)	2.80 (5)	3.317 (8)	122 (4)
C6—H6···S1	0.93	2.57	3.264 (3)	132
C4—H4···Cg2^iv^	0.93	2.81	3.599 (3)	143

Symmetry codes: (i) −*x*, −*y*+2, −*z*; (ii) *x*, *y*−1, *z*; (iii) −*x*, *y*−1, −*z*+1/2; (iv) −*x*+1/2, *y*−1/2, −*z*+1/2.
